# Supplementary material for: LNX1 Modulates Notch1 Signaling to Promote Expansion of the Glioma Stem Cell Population during Temozolomide Therapy in Glioblastoma
Source: Cancers (Basel). 2020 Nov 25;12(12):3505. doi: 10.3390/cancers12123505 (PMC7759984; doi:10.3390/cancers12123505)
Supplement: Supplementary file 1 [file cancers-12-03505-s001.pdf]

Supplementary Materials:

LNX1 Modulates Notch1 Signaling to Promote Expansion of the Glioma Stem Cell Population During Temozolomide Therapy in Glioblastoma

Shivani Baisiwala, Robert R Hall III, Miranda R Saathoff, Jack M Shireman, Cheol Park, Shreya Budhiraja, Chirag Goel, Louisa Warnke, Clare Hardiman, Jenny Y Wang, Katy McCortney, Craig M. Horbinski, Atique U. Ahmed

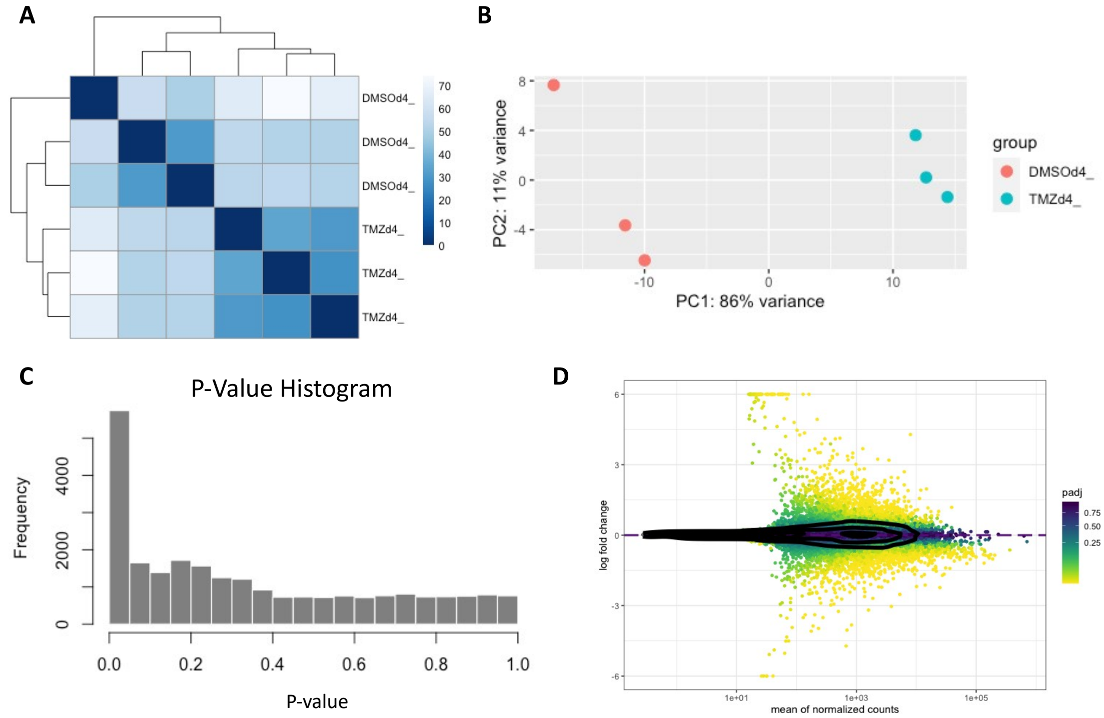

Figure S1. RNA-Seq Quality Metrics. (**A**) Sample distance clustering shows that replicates are more similar to each other and that there are differences between our control and treatment groups. (**B**) PCA shows that the greatest element of difference between our samples comes from the treatment performed. (**C**) Waterfall plot of p-values shows that we have a number of genes that were significantly altered **(D**) MA-plot represents appropriate shape for trustworthy RNA-Seq data.

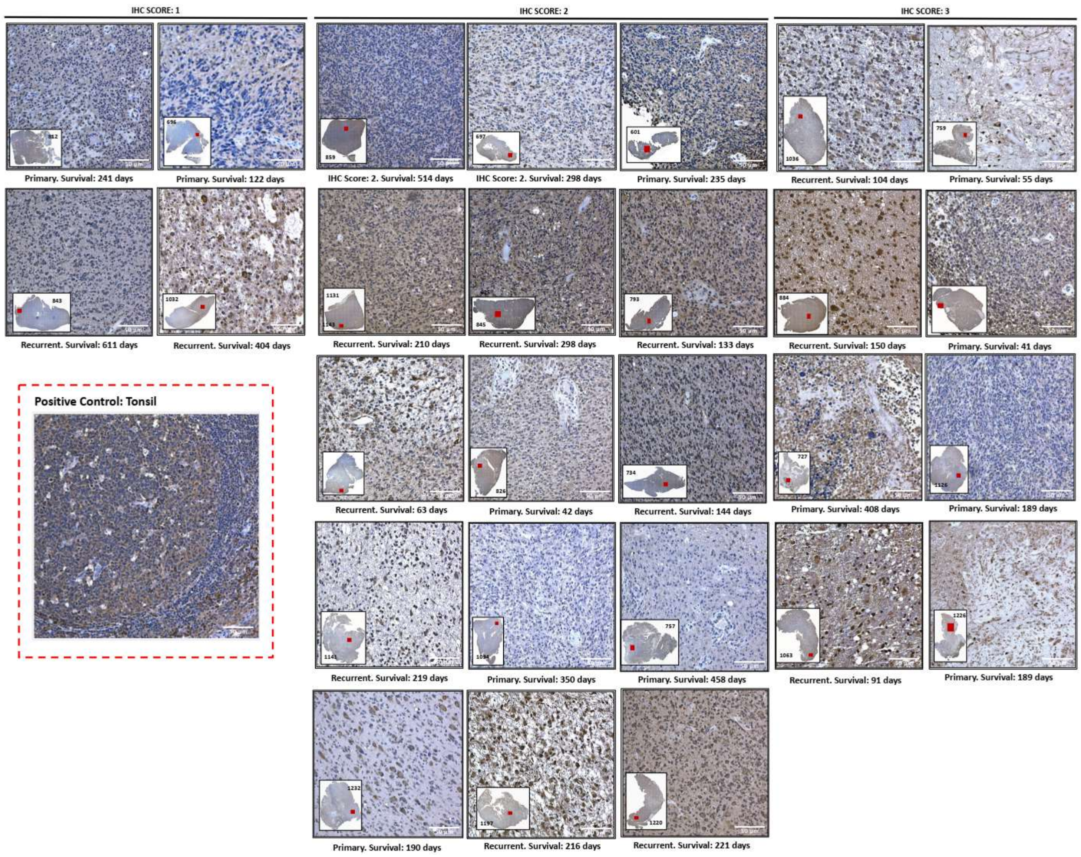

Figure S2. LNX1 Expression in Patient Samples**.** Images represent immunohistochemistry against LNX1 in GBM patient samples isolated from consenting patients at our institution. Samples were scored by a board-certified neuropathologist and de-identified survival data was correlated with the scores. Positive control for staining was tonsillar tissue, which is known to express LNX1 at high levels.

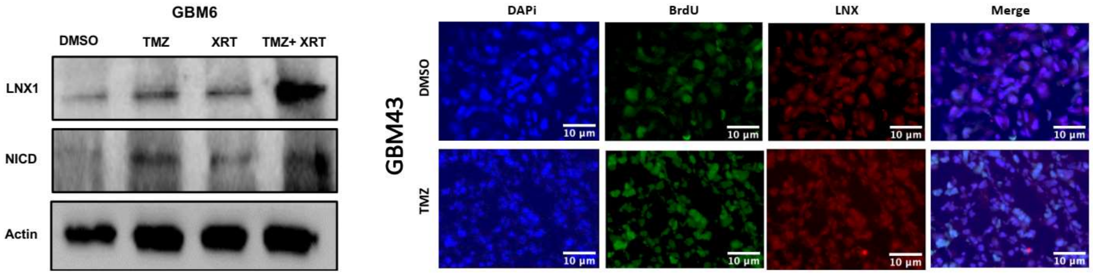

Figure S3. LNX1 Expression Elevates After Exposure to Therapeutic Stress**.** (**A**) LNX1 and NICD levels were assessed by western blot after exposure to TMZ, radiation, and combination therapy. Results showed that both were elevated upon exposure to therapeutic stress. (**B**) GBM43 cells were implanted intracranially to generate an animal tumor model. Tissues were then sectioned and stained for LNX1 and the proliferation marker BrdU. Increased expression of both is noted in tumor areas after TMZ therapy as compared to control DMSO.

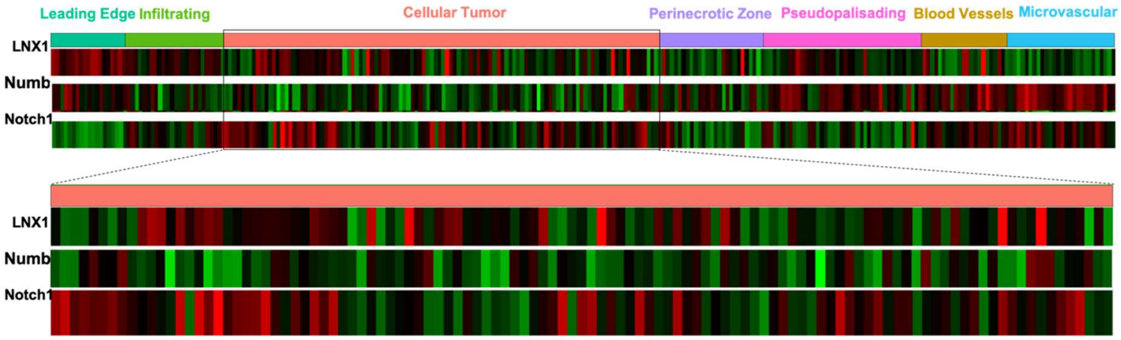

Figure S4. IvyGap provides in situ RNA hybridization data showing expression of genes within various regions of patient tumors. The cellular tumor was assessed for LNX1, Numb, and Notch1 expression and reflected that LNX1 was elevated, corresponding with Numb loss, and Notch1 elevations.

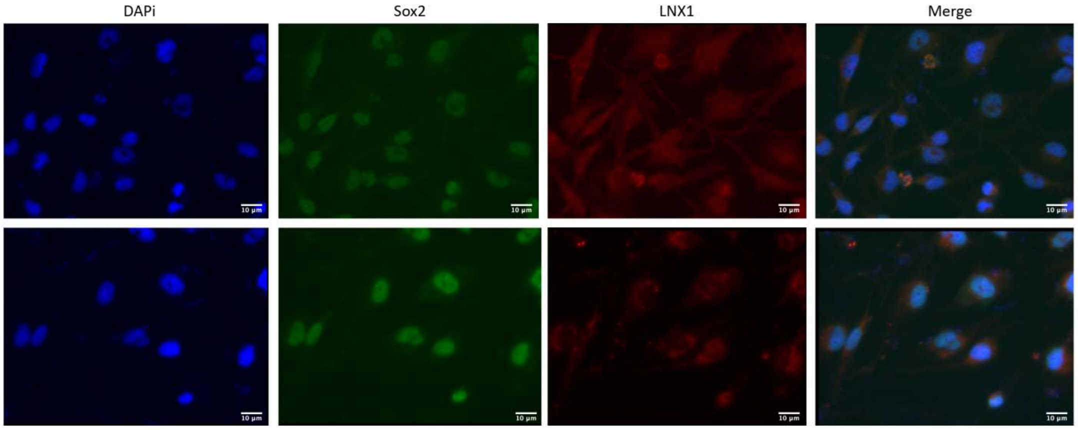

Figure S5. Cell identity confirmation was performed by immunostaining for LNX1 and Sox2, a well-known stem cell marker. Results showed that cells expressing LNX1 consistently expressed Sox2, thus confirming their identity as GSCs.

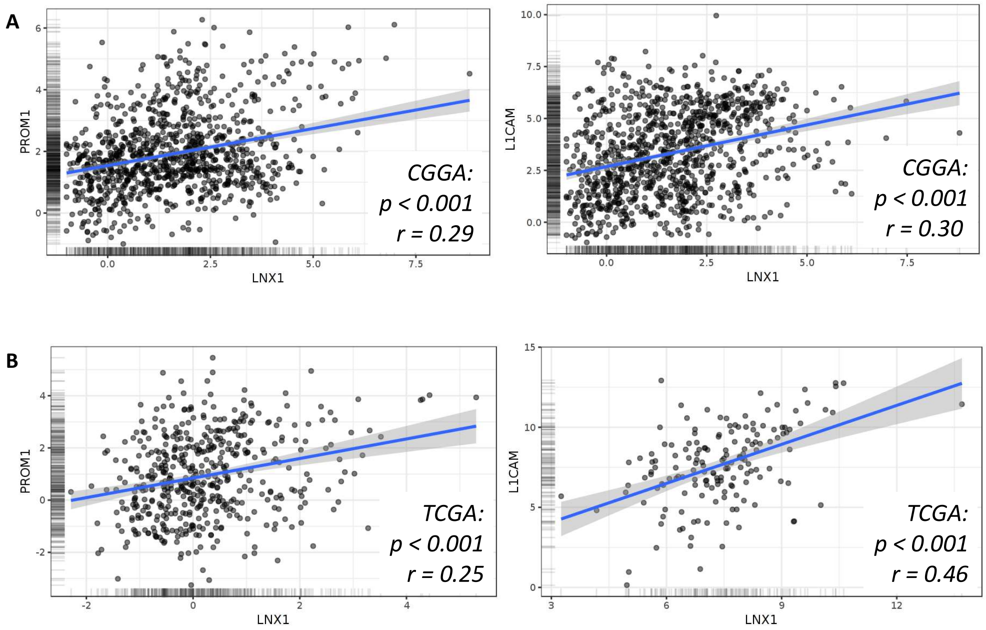

Figure S6: GlioVis datasets CGGA and TCGA were examined for correlations between LNX1 and GSC markers CD133 (PROM1) and L1CAM. (**A**) In the CGGA dataset, we noted positive correlations between LNX1 and PROM1/L1CAM. (**B**) In the TCGA_GBM dataset, we noted positive correlations between LNX1 and PROM1/L1CAM

Original Western Blot Figures

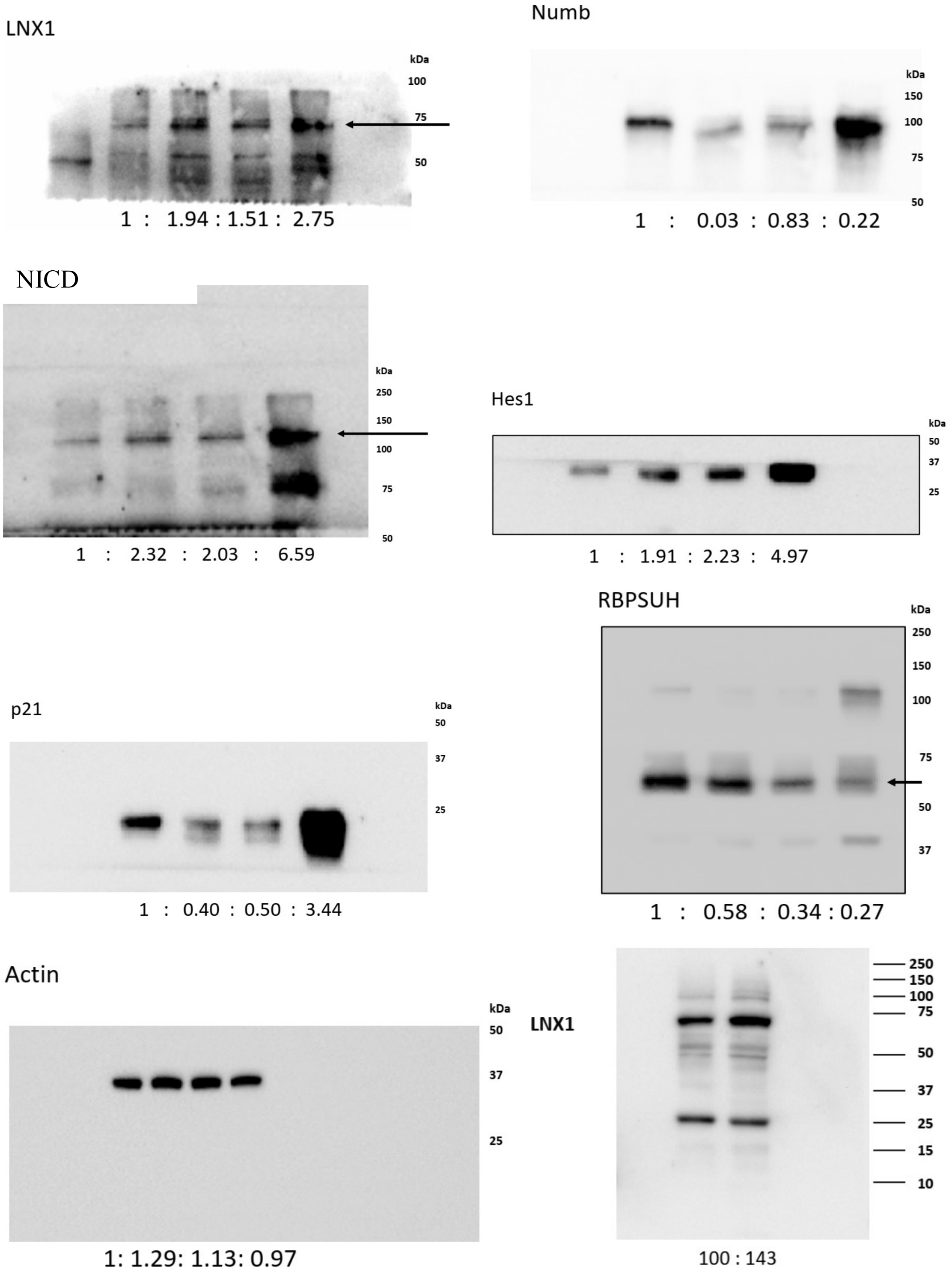

Numb

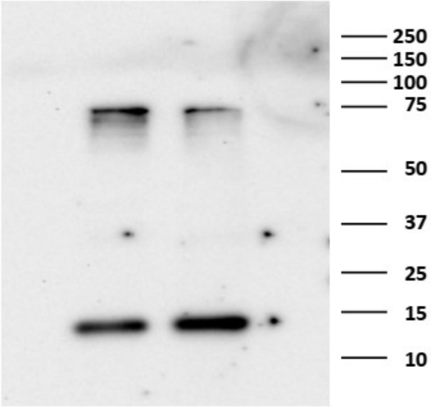

100 : 53

NICD

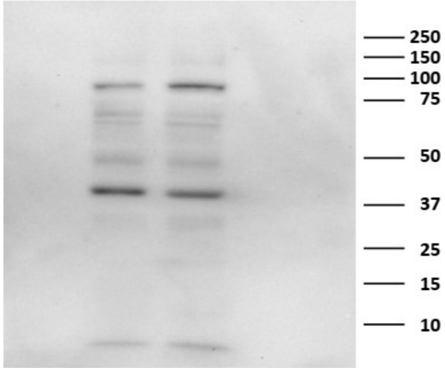

100 : 157

Hes1

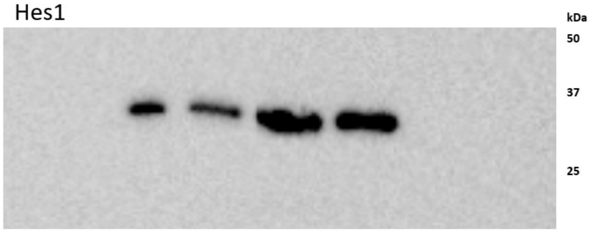

1 : 0.83 : 1.14 : 1.16

p21

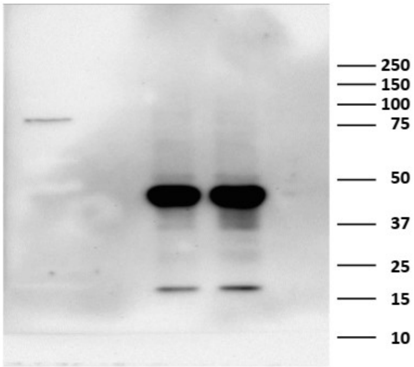

100 : 150

b-Actin

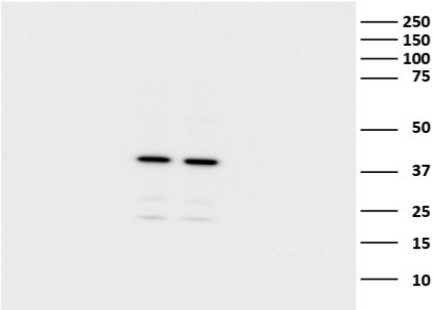

100 : 101

LNX1

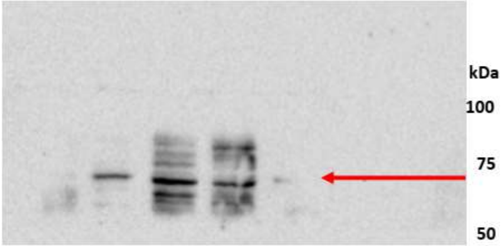

1: 1.28: 0.55: 0.18

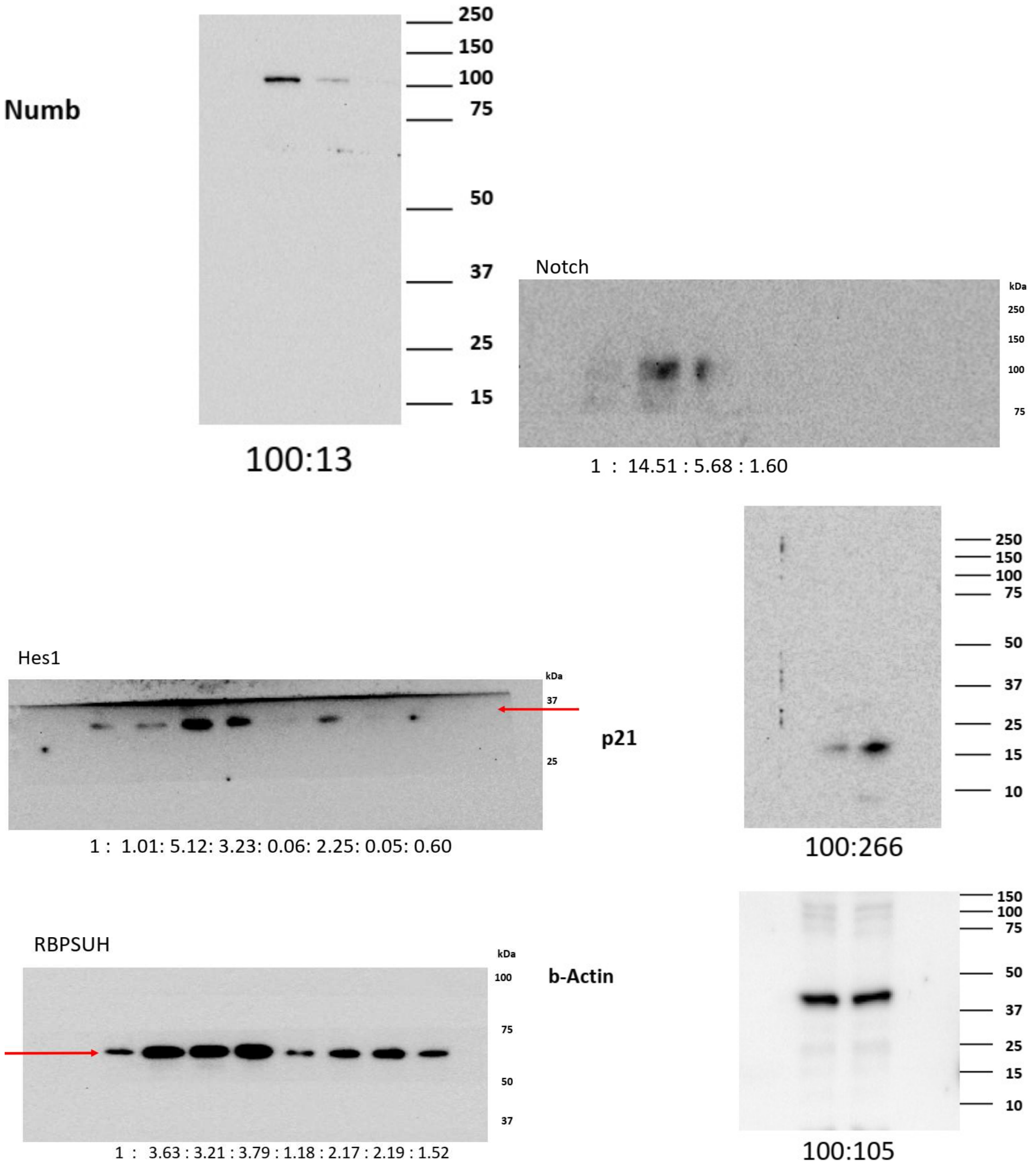

Numb
250
150
100
75
50
37
25
15
100:13
Notch
kDa
250
150
100
75
1 : 14.51 : 5.68 : 1.60
Hes1
kDa
37
25
1 : 1.01: 5.12: 3.23: 0.06: 2.25: 0.05: 0.60
p21
250
150
100
75
50
37
25
15
10
100:266
RBPSUH
kDa
100
75
50
37
1 : 3.63 : 3.21 : 3.79 : 1.18 : 2.17 : 2.19 : 1.52
b-Actin
150
100
75
50
37
25
15
10
100:105

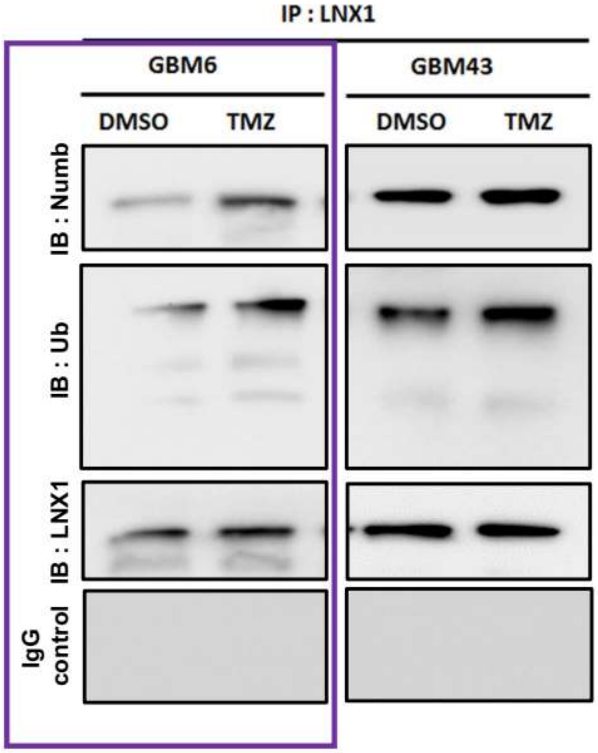
IP : LNX1
GBM6
GBM43
DMSO
TMZ
DMSO
TMZ
IB : Numb
IB : Ub
IB : LNX1
IgG control

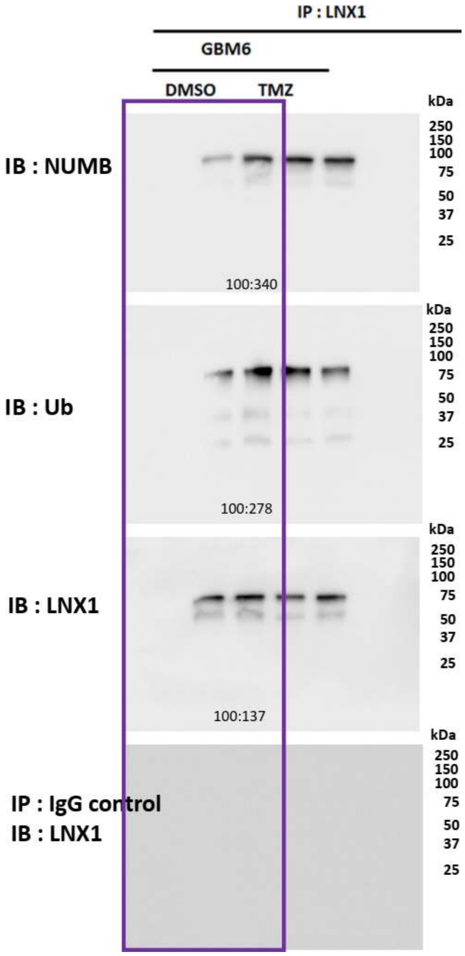
IP : LNX1
GBM6
DMSO
TMZ
kDa
250
150
100
75
50
37
25
IB : NUMB
100:340
IB : Ub
100:278
IB : LNX1
100:137
IP : IgG control
IB : LNX1

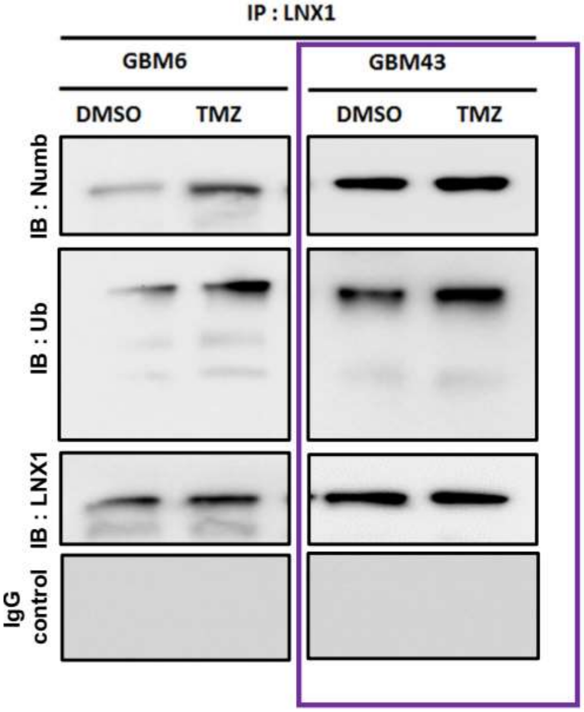
IP : LNX1
GBM6
GBM43
DMSO
TMZ
DMSO
TMZ
IB : Numb
IB : Ub
IB : LNX1
IgG control

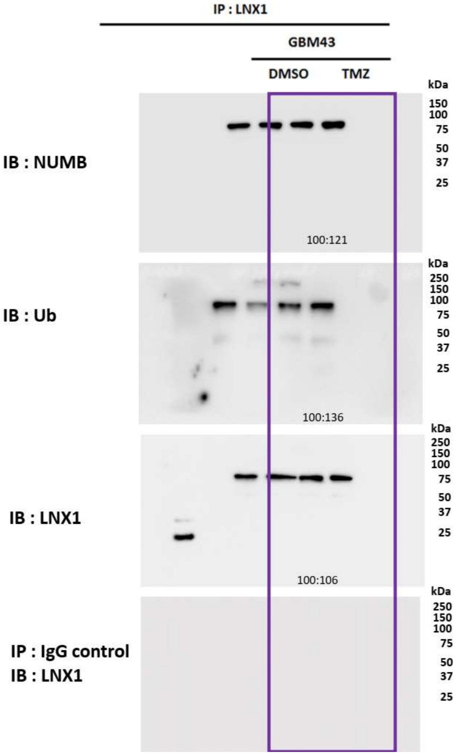
IP : LNX1
GBM43
DMSO
TMZ
kDa
250
150
100
75
50
37
25
IB : NUMB
100:121
IB : Ub
100:136
IB : LNX1
100:106
IP : IgG control
IB : LNX1

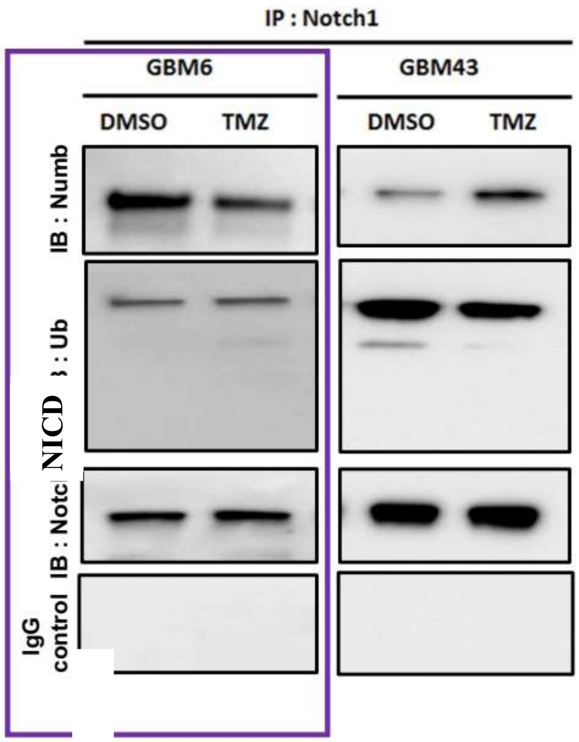
IP : Notch1
GBM6
GBM43
DMSO
TMZ
DMSO
TMZ
IB : Numb
IB : Ub
NICD
IB : Notc
IgG control

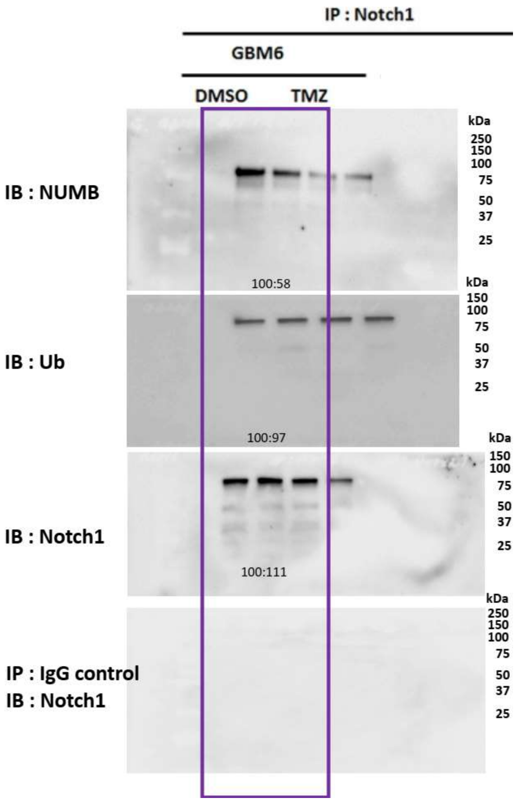
IP : Notch1
GBM6
DMSO
TMZ
IB : NUMB
IB : Ub
IB : Notch1
IP : IgG control
IB : Notch1
kDa
100:58
100:97
100:111

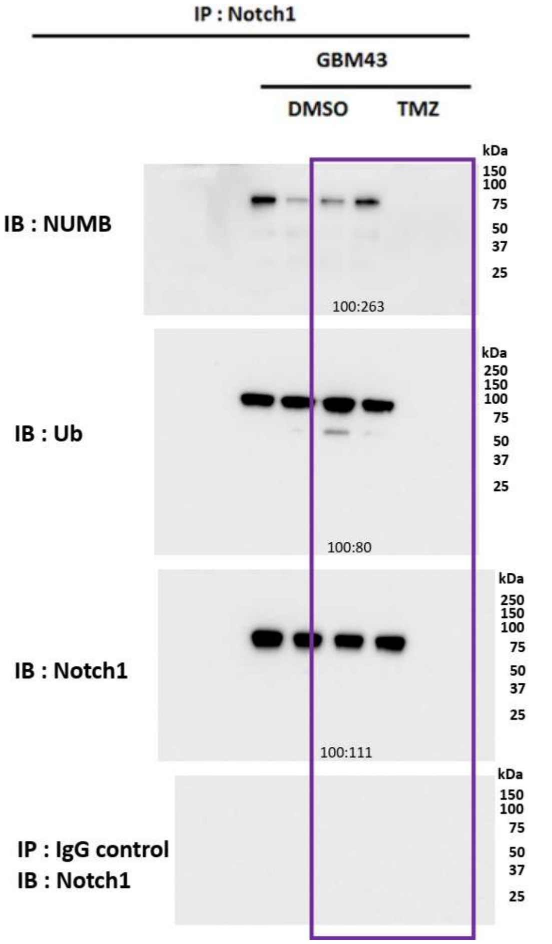
IP : Notch1
GBM43
DMSO
TMZ
IB : NUMB
IB : Ub
IB : Notch1
IP : IgG control
IB : Notch1
kDa
100:263
100:80
100:111

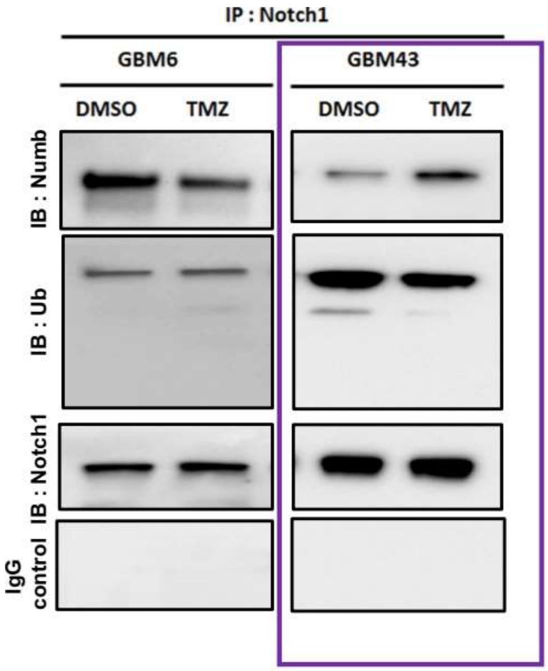
IP : Notch1
GBM6
GBM43
DMSO
TMZ
DMSO
TMZ
IB : Numb
IB : Ub
IB : Notch1
IgG control

Notch1

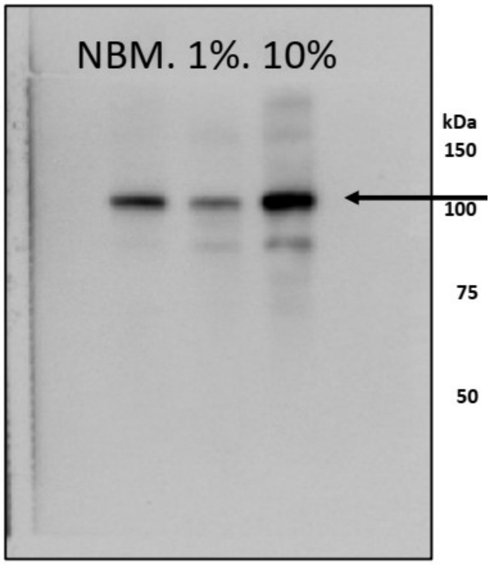

1 : 0.65 : 1.73

LNX1

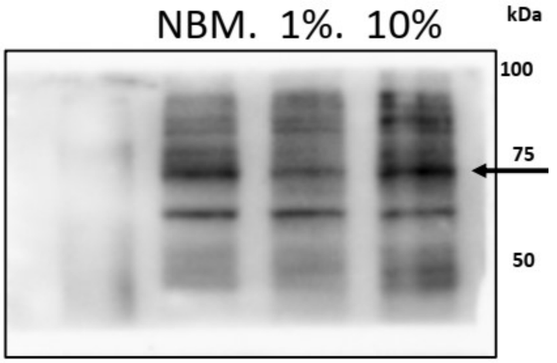

1 : 0.26 : 0.76

Actin

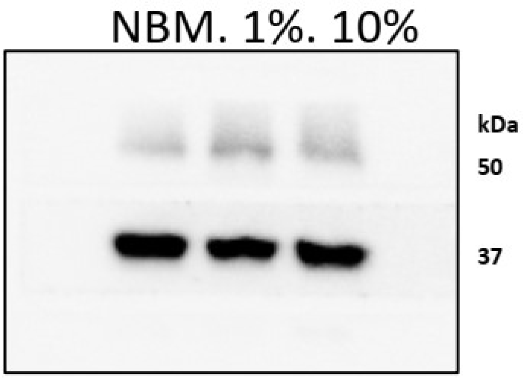

1 : 0.85: 0.92

Numb

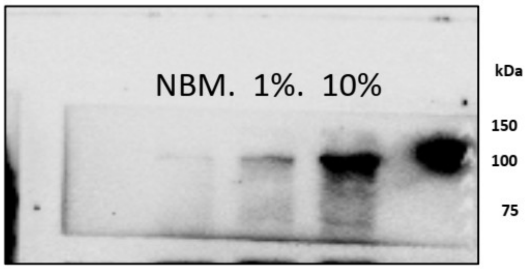

1 : 18.91 : 99.21

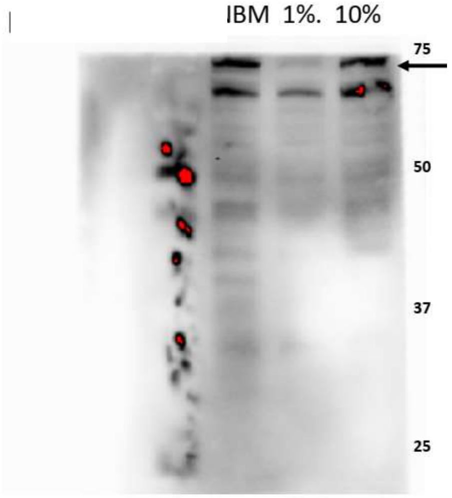

1 : 0.33: 0.62

Notch1

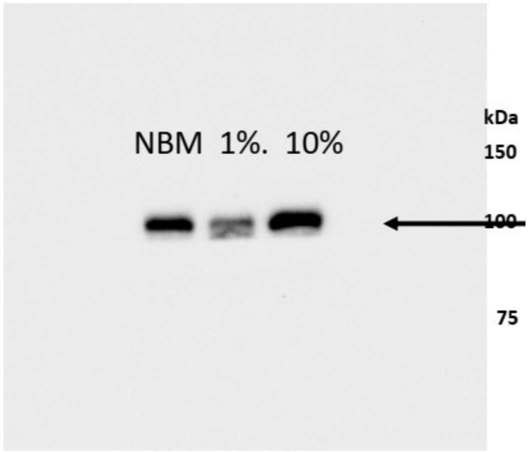

1 : 0.65: 1.73

Numb

GBM5 – 6 – 43 (NBM – 1% for each) – .

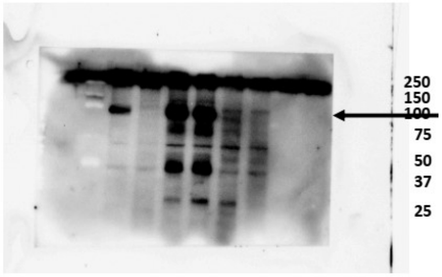

1: 0.99: 20.79: 26.44: 1.25: 2.31

Actin

NBM 1%. 10%

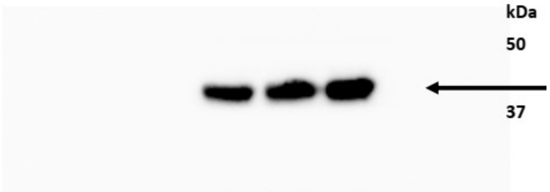

1 : 1.18: 1.39

LNX1

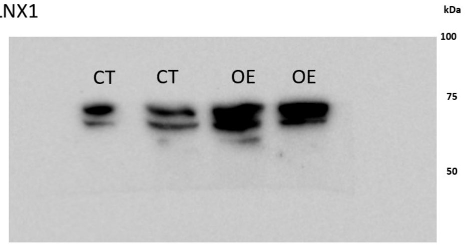

1 : 1.60 : 2.78 : 2.27

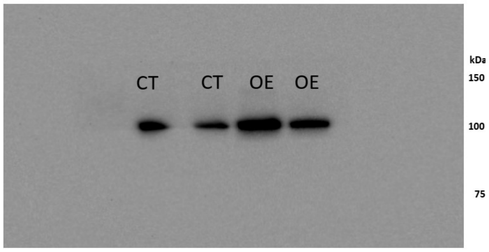

1 : 0.84 : 2.01 : 1.40

Numb

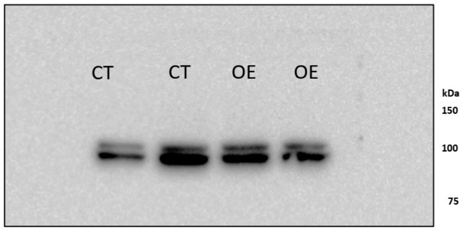

1 : 1.79 : 1.66 : 1.26

Actin

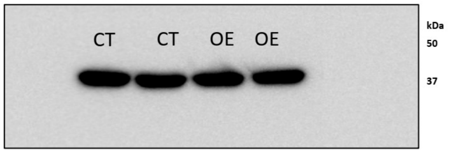

1 : 0.93 : 0.95 : 0.96

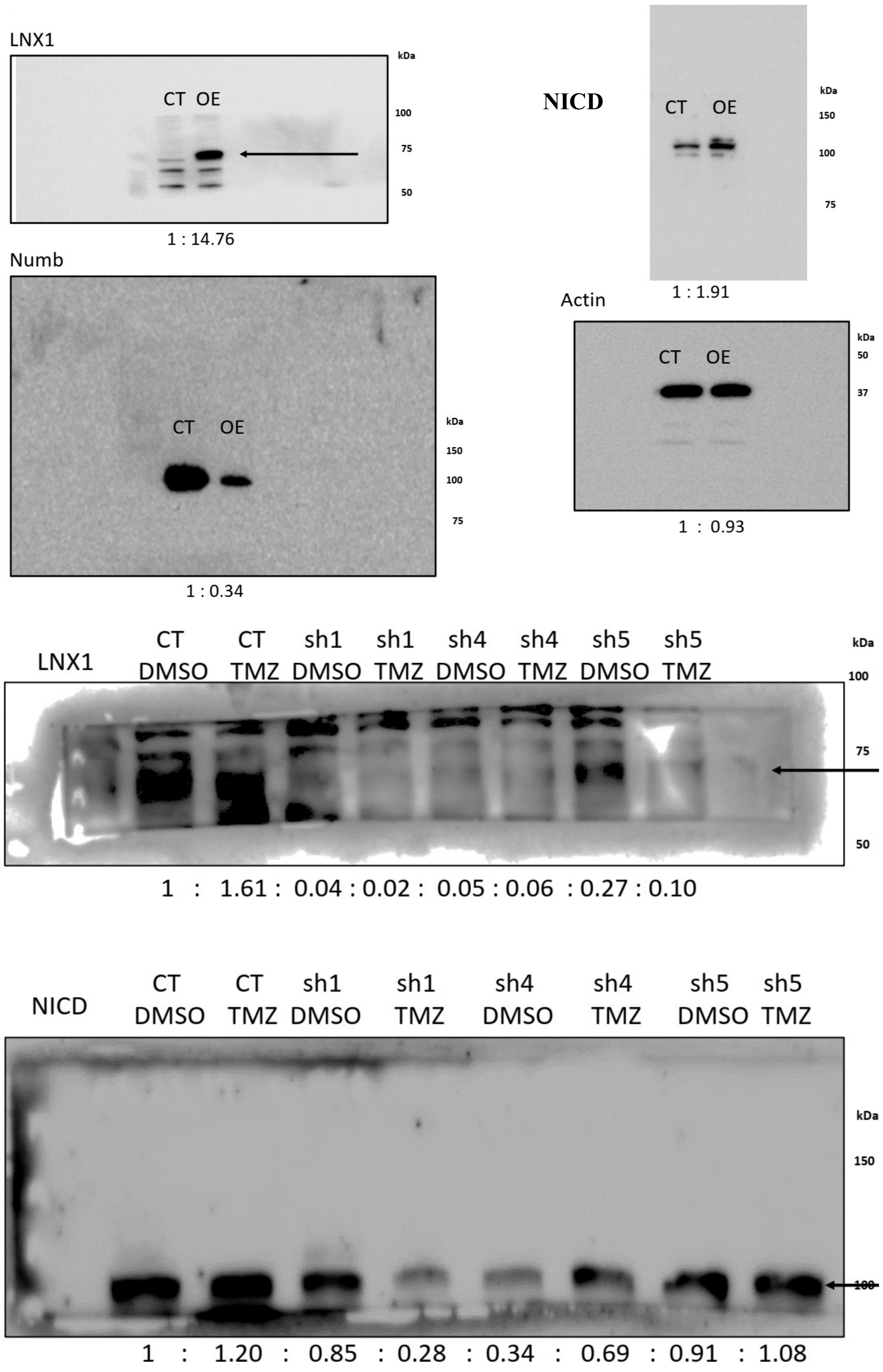
LNX1
CT OE
kDa
100
75
50
1 : 14.76
NICD
CT OE
kDa
150
100
75
1 : 1.91
Numb
CT OE
kDa
150
100
75
1 : 0.34
Actin
CT OE
kDa
50
37
1 : 0.93
LNX1
CT DMSO
CT TMZ
sh1 DMSO
sh1 TMZ
sh4 DMSO
sh4 TMZ
sh5 DMSO
sh5 TMZ
kDa
100
75
50
1 : 1.61 : 0.04 : 0.02 : 0.05 : 0.06 : 0.27 : 0.10
NICD
CT DMSO
CT TMZ
sh1 DMSO
sh1 TMZ
sh4 DMSO
sh4 TMZ
sh5 DMSO
sh5 TMZ
kDa
150
100
1 : 1.20 : 0.85 : 0.28 : 0.34 : 0.69 : 0.91 : 1.08

Numb (lanes are as above)

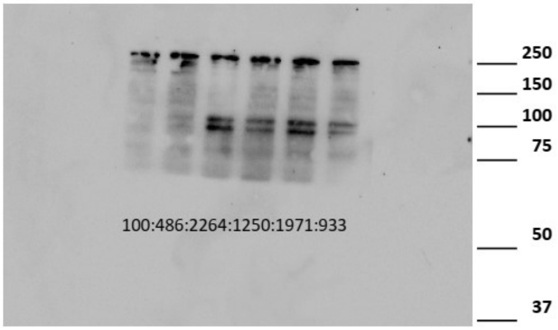

Actin

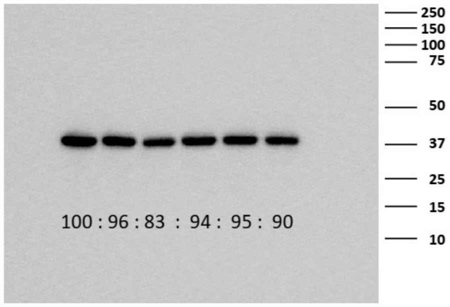

NICD

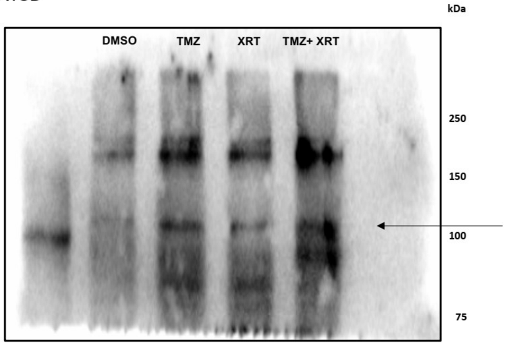

1 : 2.42 : 1.81 : 2.55

LNX1

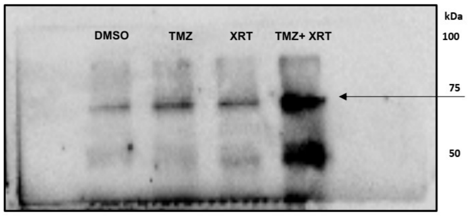

1 : 2.14 : 1.85 : 4.11

Actin

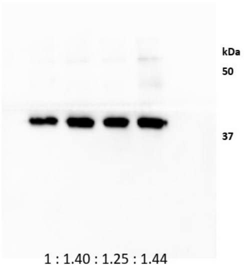

Numb

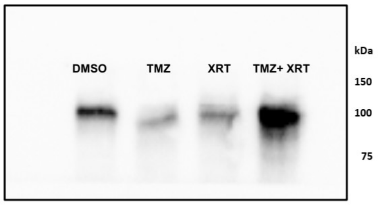

1 : 0.29 : 0.50 : 2.00
